# Temporal and spatial changes and factors influencing the industrial structure resilience of resource-based cities in western China

**DOI:** 10.1371/journal.pone.0306610

**Published:** 2024-08-14

**Authors:** Hanwen Wu, Rongguang Zhang

**Affiliations:** School of Management Science, Chengdu University of Technology, Chengdu, China; Sapienza University of Rome, ITALY

## Abstract

Due to the impact of natural disasters and public security incidents, the industrial chain is broken, and the economy has declined, especially in western resource-based cities of China where enterprises have closed down, the unemployed have increased sharply, and social contradictions have become prominent. Therefore, the resilience of the industrial structure is an urgent problem to solve in the academic circle and sustainable development. This paper identifies the spatio-temporal evolution characteristics of the resilience of the resource-based cities’ industrial structure in western China from 2006 to 2021 to provide a reference for the improvement of their industrial structure resilience.

## 1. Introduction

### 1.1 Research background

Currently, the global economic environment is complex, and natural disasters and public security incidents frequently occur, especially the COVID-19 pandemic, which has caused a continuous impact on the global economy. China’s economic development is facing the triple pressure of demand contraction, supply shock, and weakening expectations (Li 2022) [1, 2]. It is urgent to improve China’s economic resilience to stabilize economic operation, but economic resilience is closely related to the resilience of the industrial structure. The 2022 “Two Sessions” work report emphasizes that stability should be the top priority in this year’s work, and progress should be made while maintaining stability. A series of macro-control policies to stabilize the economy are an essential guarantee for enhancing the resilience of China’s economy. Improving the industrial structure’s resilience is also an inexhaustible source of driving force for China’s economy to seek change and innovate while maintaining stability. However, due to the vast territory of China and the influence of different resource endowments, development foundations and paths, and the high-quality development of China’s economy inevitably have spatial differences, and the resilience of China’s industrial structure also inevitably has spatial differences [3, 4]. The unique resource-based industries formed by resource-based cities relying on the natural resource endowment of the region are an important support for the region’s sustainable and healthy economic development, which has also made inalienable contributions to China’s economic development. However, in the era of green development and high-quality development, resource-based cities in western China have become a weak link in economic development. Therefore, accelerating the transformation of industrial structure and making the industrial structure more resilient have become the top priority for the high-quality development of western resource-based cities. Then, the scientific evaluation, differences and evolution characteristics, and the main factors affecting the improvement of the resilience of the industrial structure of western resource-based cities are discussed. It provides a decision-making basis for realizing high-quality urban transformation and development.

Industrial structure plays a crucial role in the economic development and environmental sustainability of a country. Several studies have explored the relationship between industrial structure and various factors such as environmental regulations, land transfer marketization, green credit, carbon emissions, energy endowment, higher education development, technological innovation, and pilot programs for low-carbon cities. Wu et al. (2021) delved into the relationships among carbon emissions, industrial structure, economic growth, energy endowment, and CO2 emissions in China [5]. Dong et al. (2020) also discussed achieving a win-win situation between economic growth and carbon emission reduction through industrial structure upgrading [6]. Wu et al. (2021) revisited the resource curse in the context of carbon emissions by analyzing the effects of energy endowment and industrial structure upgrading on carbon emissions. Furthermore, Wu et al. (2021) highlighted the positive spatial spillover effects of higher education development and technological innovation on industrial structure upgrade [7]. Zheng et al. (2021) examined the impact of pilot programs for low-carbon cities on industrial structure upgrading, providing insights for integrated development [8]. Song et al. (2021) explored the influence of environmental regulations on industrial structure upgrading, emphasizing the strategic interaction behavior among local governments [9]. Overall, these studies underscore the importance of industrial structure optimization, environmental regulations, green credit, and other factors in achieving sustainable economic growth and environmental protection in China.

The concept of resilience was introduced in the paper “Resilience and Stability of Ecological Systems,” published by Holling [10] in 1973. It was the first time researchers applied the concept of “resilience” to ecology. Since then, research and practice on resilience have emerged in many fields and disciplines globally. The word “resilience” has thus become an important research entry point and practice combination point in academia and all sectors of society [11, 12]. Martin (2012) defined economic resilience as the economic system showing specific stability in the face of external shocks and adapting to and transitioning to a new steady state [13]. Based on this definition, Sunley (2015) divided economic resilience into four dimensions: resistance, resilience, reorganization, and renewal [14]. Cao Guangzhong (2021) believed that the industrial structure of a city is one of the most critical factors affecting the resilience of the urban economy, and the degree of industrial diversification and specialization and the proportion structure of different industries in a region will also affect the resilience of the industrial structure itself [15]. For the current measurement method of industrial structure resilience, Li Liangang (2019), a domestic scholar, decomposed regional economic resilience into an industrial structure component and a regional competitiveness component with the shift-share analysis method to reveal the mechanism of regional economic resilience [16]. Guan Haoming (2021) considered the financial crisis and other shocks as the background, selected core variables such as employment or gross domestic product (GDP), and analyzed the characteristics of Shenyang’s economic resilience since 1978 from the perspectives of macroeconomic increment, meso industrial increment and structural change, and micro-enterprise spatial dynamics [17]. However, the most mainstream calculation method is based on the single indicator calculation method proposed by Martin (2015), that is, measuring the resilience of industrial structure by showing the response degree of the regional economy to shocks through the number of employees or GDP, and the indicator system method. Cai Jing (2023) has comparatively analysed the social, economic and environmental development of Fujian, Guangdong and Zhejiang before and after covid-19, using data collected from relevant economic and social sectors in China. Recommendations are made for the future social, economic and environmental development of the study areas [18]. The literature on the resilience of industrial structures encompasses a variety of perspectives and methodologies. Pai (2016) focuses on the total factor productivity (TFP) growth of targeted industries in Korea, highlighting the importance of institutions and industrial policies for industrial upgrading [19]. Ashton et al. (2017) explore the dynamics of industrial ecosystems, emphasizing the material and socio-economic aspects of connectivity between firms to determine resilient structures [20]. Guan et al. (2018) analyze the industrial structure evolution of old industrial cities in China using evolutionary resilience theory, providing insights for revitalization efforts [21]. Tan et al. (2020) investigate the role of industrial structure in regional economic resilience in resource-based cities in China, emphasizing the need for a multi-scalar and contextually sensitive perspective [22]. Chen et al. (2022) examine the relationship between industrial structure, environmental pressure, and ecological resilience in resource-based cities in China, highlighting the importance of promoting green development [23]. Sutton et al. (2022) study the resilience of Canadian cities during the 2020 economic crisis, finding that local-specific effects play a dominant role [24]. Duan et al. (2022) emphasize the impact of industrial structure on economic resilience [25]. Lee et al. (2023) conduct an empirical analysis on the economic resilience of industrial parks in Taiwan, emphasizing adaptability, innovation, and sustainability [26]. He et al. (2023) investigate the mechanism and impact of the digital economy on urban economic resilience in China, proposing suggestions for digital development and talent training [27]. Lu et al. (2024) consider the moderating role of industrial structure in the effects of environmental regulation policy synergy on ecological resilience, finding varying effects across different quantiles [28]. Overall, these studies contribute to a nuanced understanding of the factors influencing the resilience of industrial structures in various contexts.

### 1.2 Research significance

Some scholars have begun to pay attention to the measurement of economic resilience of special types of regions in China, such as contiguous poverty-stricken areas and old industrial bases. In terms of the analysis of influencing factors, domestic research mainly focuses on the impact of economic agglomeration, industrial diversification, innovation, and manufacturing development on economic resilience. Some scholars pay attention to the impact of a certain shock on economic resilience. For example, Liu Chengkun studied the impact of industrial structure diversification on the economic resilience of Guangdong-Hong Kong-Macao Greater Bay Area under the public health emergency. Wang Yonggui qualitatively studied the relationship between the impact of COVID-19, economic resilience, and China’s high-quality development [29]. Based on understanding the impact characteristics and development trend of COVID-19, Li Liangang measured China’s provincial economic resilience from the perspective of resistance, and analyzed the influencing factors causing the spatial-temporal differences in economic resilience. Wang Xinyuan quantitatively diagnosed the main obstacle factors affecting the economic resilience of resource-based cities in the Yellow River Basin from three aspects: the ability to resist risks, the ability to adapt to development, and the ability to innovate and transform. Resource-based cities in China have been the focus of various studies in recent years. Li et al. (2013) highlighted that these cities were less developed compared to others, prompting the need for economic transition policies [30]. He et al. (2017) delved into the economic restructuring of mining cities in China, emphasizing the importance of policy-making for resource-based cities in developing countries [31]. Chen et al. (2018) evaluated the economic transformation and upgrading of resource-based cities in Shaanxi Province, utilizing an improved TOPSIS method [32]. Yang et al. (2019) conducted a regional analysis of green development levels in Chinese mineral resource-based cities, constructing a comprehensive evaluation index system [33]. Chen et al. (2019) explored the industrial land use efficiency of China’s resource-based cities using Data Envelope Analysis (DEA) and the Tobit model to analyze influencing factors [34]. Yan et al. (2019) focused on urban sustainability in Chinese resource-based cities, highlighting the determinants of total factor energy efficiency and the impact of government intervention [35]. Moreover, Song et al. (2020) conducted a study on the impact of low-carbon city construction on ecological efficiency in China, showing significant improvements in urban ecological efficiency due to low-carbon city pilot policies [36]. Li et al. (2020, 2021) examined the effects of China’s Sustainable Development Plan for Resource-based Cities on local industrial transformation, finding a reduction in the proportion of secondary industries in GDP [37, 38]. Fan et al. (2021) analyzed the transformation effect of resource-based cities based on the PSM-DID model, evaluating the impact of the National Plan for Resource-based City Sustainable Development on city characteristics over a ten-year period [39]. Overall, these studies provide valuable insights into the economic, environmental, and social aspects of resource-based cities in China, highlighting the importance of policy interventions and sustainable development strategies to promote growth and transformation in these cities.

While most studies on industrial structure resilience in domestic settings predominantly focus on macro levels [40] such as city clusters and provincial areas, this paper takes a more granular approach by examining the industrial structure of individual cities. Specifically, the research delves into the industrial structure of western resource-based cities, evaluating their industrial composition, level of advanced industrialization, and overall industrial development. By comparing the differences of cities before and after the impact of the covid-19, the study assesses the resilience of the region’s industrial structure. This analysis aims to identify key influencing factors and recommend corresponding measures to enhance the industrial structure resilience of western resource cities. Moreover, this research contributes to expanding the scope and depth of future studies on industrial structure transformation in resource-rich areas.

## 2. Study area and data sources

### 2.1 Area of study

**Fig 1:**
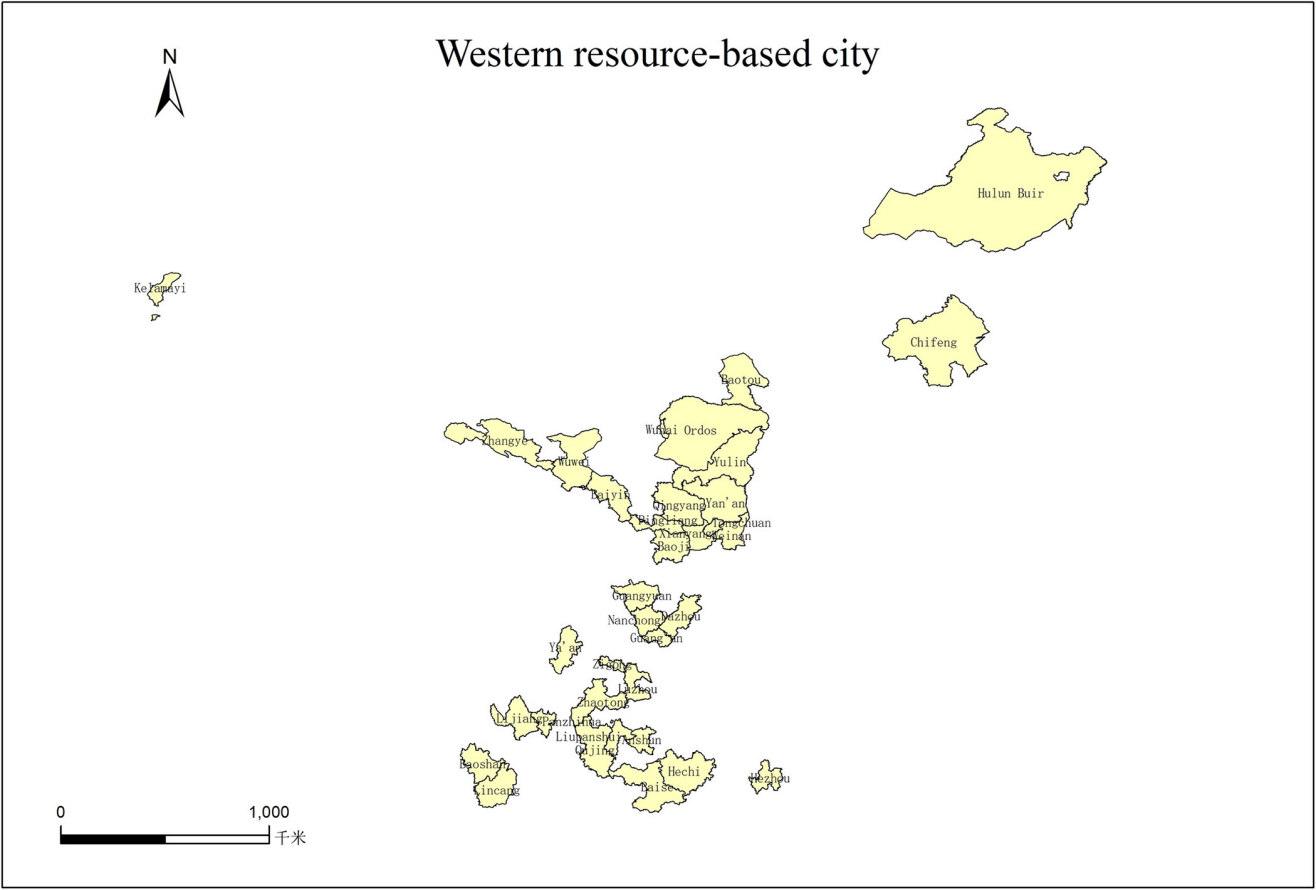
Western resource-based city.

Since the middle of the last century, resource-based cities have emerged in China. In the decades after the founding of the People’s Republic of China, China achieved rapid economic growth based on increasing the output value of the heavy industry, and the output of resource-based cities made important contributions to the economic growth during this period. In the National Sustainable Development Plan for Resource-based Cities (2013–2020) (hereinafter referred to as the Plan) issued in 2013, it is pointed out that there are 262 resource-based cities in China [41], among which 102 are in the western region, accounting for about 39% of the total. In addition, according to the development stage, China’s resource-based cities are further divided into four categories [42]: growth, maturity, decline, and regeneration, accounting for 37.5%, 43.75%, 10.41%, and 8.3% of the resource-based cities above prefecture-level in western China, respectively. Considering the availability of data, 11 cities, prefectures-districts with missing data are excluded, namely Liangshan Yi Autonomous Prefecture, Haixi Mongolian and Tibetan Autonomous Prefecture, Chuxiong Yi Autonomous Prefecture, Qiannan Buyi and Miao Autonomous Prefecture, Bayingoling Mongolian Autonomous Prefecture, Qianxinxi Buyi and Miao Autonomous Prefecture, Aba Tibetan and Qiang Autonomous Prefecture, Pu’er City, Bijie City, Jinchang City, and Altay Prefecture [43, 44]. Other 38 cities are selected as the research area of this paper, As shown in Table 1 and Fig 1.

**Table 1:** Distribution of 38 resource-based prefecture-level administrative regions in western China.

Province of residence (District and city)	Prefecture-level administrative region
**Inner Mongolia (5)**	Baotou、Wuhai、Chifeng、Hulunbeier、Erdos
**Guangxi (3)**	Baise、Hechi、Hezhou
**Sichuan (8)**	Guangyuan、Nanchong、Guangan、Zigong、Luzhou、Panzhihua、Dazhou、Ya’an
**Guizhou (3)**	Liupanshui、Anshun、Bijie
**Yunnan (6)**	Qujing、Baoshan、Zhaotong、Lijiang、Lincang、Puer
**Shanxi (6)**	Yan’an、Tongchuan、Weinan、Xianyang、Baoji、Yulin
**Gansu (5)**	Baiyin、Wuwei、Zhangye、Qingyang、Pinliang
**Ningxia (1)**	Shizuishan
**Xinjiang (1)**	Kelamayi

### 2.2 Construct index system and data source

Currently, the methods for measuring economic resilience mainly include the single-indicator calculation method proposed by Martin, that is, measuring economic resilience by showing the degree of regional response to economic shocks, such as the number of employees or GDP, and the indicator system method. Although the index system method still has defects, it can better reflect the different dimensions of economic resilience and the characteristics of resource-based cities by establishing an index system. Martin believes that economic resilience includes four dimensions: Resistance, the response of the economic system to shocks; Resilience, the speed and state of recovery after a shock; Reconfiguration, the ability of an economic system to redefine and adapt to new circumstances in response to shocks; and Renewal power, the ability of a regional economy to reconstruct its pre-shock growth path or to choose a new path after an economic shock. The reconstruction and update forces are relatively similar in data selection. Therefore, based on the existing experience, this paper will build a comprehensive evaluation index system from three aspects: the ability to resist risks, adapt to development, and innovate and transform. The index system is constructed for the specific index selection by referring to the existing indicators in relevant domestic and foreign literature [11] and combining them with the characteristics of resource-based cities [40], as shown in the Table 2.

**Table 2:** Comprehensive index system.

Objective level	first level indicator	second level indicator	Index property
Resilience of industrial structure	Ability to resist risk	GDP per capita	+
		Proportion of employment in mining	-
		Proportion of employees in state-owned units	-
		Per capita disposable income of urban residents	+
		The proportion of tertiary industry in GDP	+
		Foreign trade dependence	-
		Resource contribution	+
		Market allocative efficiency	+
	Ability to adapt to development	Self-financing level of local governments	+
		Fixed asset investment growth rate	+
		Total retail sales of social goods	+
		Energy consumption reduction rate of RMB 10,000 regional GDP	+
		Regional GDP growth rate	+
		Registered urban unemployment rate at year-end	-
	Innovation and transformation capability	Industrial upgrading	+
		Other resource support and supply degree	+
		The decrease rate of personnel in state-owned units	+
		The proportion of employment in mining industry decreased	+
		Scientific research investment	+
		Investment in education	+

The data in this paper mainly come from the 2013–2021 China City Statistical Yearbook, the City Statistical Yearbooks of each research object, and their statistical bulletins on National Economic and Social development.

## 3. Research method

### 3.1 Global spatial autocorrelation analysis

Global spatial autocorrelation is a description of the spatial characteristics of attribute values in the whole region, which is used to analyze the overall spatial correlation of the region [45]. This paper uses Moran’s I index to judge the spatial correlation of economic resilience of resource-based cities in western China [11, 46], and the calculation formula is as follows:

$$I=\frac{n\sum_{i=1}^{n}\sum_{j=1}^{n}w_{ij}(x_{i}-\bar{x})(x_{j}-\bar{x})}{\sum_{i=1}^{n}\sum_{j=1}^{n}w_{ij}\sum_{i=1}^{n}{\left(x_{i}-\bar{x}\right)}^{2}}$$ (1)

where, I is Moran’s I index; *x_i_*、*x_j_* are the economic resilience of city i and city j, respectively; $\bar{x}$ is the mean value of economic resilience; *w_ij_* is the spatial weight matrix, which usually takes the value of 1 for adjacent units and 0 for others; n is the number of research units; the range of I is [−1,1]; the closer its absolute value is to 1, the stronger the spatial correlation is; a closer value to 0 shows a random spatial distribution.

### 3.2 Entropy method

The entropy method is a more frequently used objective weighting method. Compared with subjective weighting, the entropy method has the characteristics of high precision and strong objectivity, is very scientific to avoid the deviation of human factors, and can better explain the evaluation results. To avoid meaningless calculations in the process of using the entropy method, it is necessary to carry out dimensionless processing on the existing data, and its formula is as follows:

$$\begin{array}{l} s_{ij}=\frac{1-e_{j}}{\sum_{i=1}^{m}1-e_{j}}y_{ij}^{\prime },\\ e_{j}=-\frac{1}{\ln n}\sum_{i=1}^{n}\frac{\frac{y_{ij}^{\prime }}{\sum_{i=1}^{n}y_{ij}^{\prime }}}{n\frac{y_{ij}^{\prime }}{\sum_{i=1}^{n}y_{ij}^{\prime }}},\\ y_{ij}=\frac{x_{ij}-min(x_{ij})}{max(x_{ij})-min(x_{ij})},\\ y_{ij}=\frac{max(x_{ij})-x_{ij}}{max(x_{ij})-min(x_{ij})}, \end{array}$$ (2)

where *y_ij_* is the standardized score of the ith index; *x_ij_* is the original data value of the jth index in the ith city; *max*(*x_ij_*)、*min*(*x_ij_*) are the maximum and minimum original values of the jth index, respectively; *e_j_* represents the entropy value of the jth index; $y_{ij}^{\prime }$ represents the value after non-zero processing; $\frac{y_{ij}^{\prime }}{\sum_{i=1}^{n}y_{ij}^{\prime }}$ represents the weight; *s_ij_* represents the evaluation score of each factor level of the ith city; thus, the evaluation score of urban economic resilience this year is *S* = ∑*s_ij_*.

### 3.3 Obstacle degree model

The main purpose of measuring the economic resilience of western resource-based cities is to reveal their economic development status in a certain period of time and their ability to resist risks to avoid one aspect becoming a weak link. A comprehensive evaluation can only measure the level of economic resilience, but cannot accurately identify the factors restricting its improvement. To this end, the obstacle degree model is introduced to explore the obstacles to improving economic resilience in western resource-based resource-based cities. Among them, factor contribution is the weight of a single index affecting the comprehensive level of economic resilience; indicator deviation degree refers to the gap between the single indicator and the target of the comprehensive level of economic resilience, that is, the difference between the evaluation value of the single indicator and 100%; and obstacle degree refers to the degree of influence of the index layer and factor layer on the comprehensive level of economic resilience. The obstacle degree model can analyze the internal factors of the system and their degree of action, fully reflect the internal law of urban economic resilience development, and then provide appropriate medicine for the obstacles. The calculation formula is as follows:

$$p_{j}=\frac{F_{j}X_{j}}{\sum_{j=1}^{n}F_{j}X_{j}}$$ (3)

In the formula, *p_j_* represents the obstacle degree of the Jth indicator to the level of economic resilience, *F_j_* represents the factor contribution degree, and *X_j_* represents the deviation degree of the indicator.

### 3.4 Industrial structure will be upgraded

According to the literature, the calculation method for the optimization of the industrial structure is as follows: the ratio of the added value of the tertiary industry to that of the secondary industry, the calculation formula is HG = Y3/Y2. Among them, HG represents the degree of industrial structure optimization, Y2 represents the added value of the secondary industry, and Y3 represents the added value of the tertiary industry. A higher value of HG indicates a more advanced industrial structure.

## 4. Results and analysis

### 4.1 Proportion of secondary and tertiary industries

The industrial structure of western resource-based regions is mainly resource-based industries, among which energy and mining industries are particularly typical and in the majority [47]. This industrial structure results in intensive employment in resource-based industries, with a large share of primary product mining, a relatively simple industrial structure, converging resources, and slow structural development. In the mining industry, the share of manufacturing in the western resource-based region is small, but the amount of mining development and primary products is excessive, thus promoting the primary product processing industry, and energy-led and mineral resources industry structure. The following analysis of the current situation of industries in western resource-based areas of China will be elaborated from the composition of GDP. As the secondary and tertiary industries account for a significant proportion of the regional GDP of most resource-based cities, the primary industry will not be discussed, and only the changes in the proportion of the secondary and tertiary industries is analyzed. The proportion of the secondary (As shown in Table 3) and tertiary industries (As shown in Table 4) can directly reflect the resilience of the region’s industrial structure.

**Table 3:** The proportion of secondary industry in regional GDP in western resource-based cities.

City	Proportion of secondary production (%)			
	2006	2011	2016	2020
Baotou	54.2	55.4	47.1	41.4
Wuhai	63.6	72.8	56.6	64.5
Chifeng	41.2	53.6	47.0	31.2
Erdos	55.0	60.1	55.7	56.8
Hulunbeier	32.2	44.5	44.7	27.9
Baise	50.2	54.5	53.4	39.8
Hezhou	46.7	46.3	40.8	34.2
Hechi	40.5	41.3	30.4	28.5
Zigong	46.1	58.8	57.5	40.1
Panzhihua	70.5	75.5	70.5	53.6
Luzhou	41.7	59.7	59.1	49.1
Guangyuan	32.7	44.7	46.6	39.0
Nanchong	38.9	50.9	46.1	37.9
Guangan	38.6	51.4	51.6	32
Dazhou	37.4	52.6	41.6	34.0
Ya’an	46.4	57.2	53.4	29.9
Liupanshui	58.9	62.7	50.2	44.8
Anshun	39.8	39.4	32.4	30.3
Qujing	53.9	53.5	49.7	37.0
Baoshan	26.6	32.1	34.9	37.3
Zhaotong	41.9	47.9	42.1	37.5
Lijiang	31.6	41.7	38.9	32.3
Lincang	33.2	38.2	33.7	24.8
Tongchuan	55.4	63.7	51.9	34.9
Baoji	58.7	63.7	63.5	55.4
Xianyang	45.0	53.4	57.9	44.1
Weinan	46.1	53.0	46.0	34.9
Yan’an	78.6	73.2	53.0	55.3
Yulin	68.2	71.1	60.6	62.5
Baiyin	54.3	57.4	40.3	34.3
Wuwei	34.9	42.3	37.0	16.2
Zhangye	35.4	37.4	27.5	18.6
Pinliang	39.4	48.3	24.8	24.5
Qingyang	57.8	63.4	48.2	47.2
Longnan	35.3	30.6	21.6	22.8
Shizuishan	64.9	64.4	63.0	47.9
Kelamayi	89.7	89.3	69.6	67.4
Total	48.7	46.8	39.8	37.8

Source: The data in the table mainly comes from the 2005–2021 China Urban Statistical Yearbook

**Table 4:** Proportion of tertiary industry in regional GDP in western resource-based cities.

City	Proportion of tertiary production (%)			
	2006	2011	2016	2020
Baotou	42.3	41.9	50.4	54.8
Wuhai	35.1	26	42.5	34.4
Chifeng	35.7	30.7	37.9	49.2
Erdos	39.7	37.3	41.9	39.4
Hulunbeier	43.4	36.8	40	47.3
Baise	27.3	26.3	30.3	40.7
Hezhou	24.6	31.5	37.6	46.7
Hechi	32.5	38.8	46.6	50.1
Zigong	34.1	28.5	31.4	45.7
Panzhihua	25	20.7	26.1	37.1
Luzhou	36.1	25.8	28.9	40.5
Guangyuan	37.9	34.6	37.3	42.5
Nanchong	33.2	25.8	30.2	42.9
Guangan	36.5	29.6	32.6	49.9
Dazhou	31.2	24.4	36.7	47.4
Ya’an	31.7	26.5	32.6	50.0
Liupanshui	33.4	32.1	40.2	42.5
Anshun	39.9	45	49.9	51.5
Qujing	27.9	27.9	42.7	44.3
Baoshan	39.2	36.9	40.4	39.6
Zhaotong	33.5	32.4	38.3	45.0
Lijiang	46.7	41.2	45.7	52.6
Lincang	30.9	29.8	38.1	29.5
Tongchuan	37	28.9	40.4	57.0
Baoji	27.8	25.3	27.6	35.6
Xianyang	32.7	27.1	27.3	40.5
Weinan	35.8	31.4	38.5	45.1
Yan’an	13.7	19	36.1	32.8
Yulin	23.8	24	33.5	30.8
Baiyin	33.3	31.4	45.7	45.5
Wuwei	39.8	33.1	39.6	53.1
Zhangye	34	34.5	46.9	53.8
Pinliang	36.8	31.1	47.2	52.4
Qingyang	25.7	23.8	37.5	39.9
Longnan	36.2	44.1	56.7	59.0
Shizuishan	29.2	29.9	39.6	45.2
Kelamayi	9.9	9.8	28.4	30.6
Total	39.5	43.1	51.6	54.5

Source: The data in the table mainly comes from the 2005–2021 China Urban Statistical Yearbook

It can be seen that during the 6 years from 2006 to 2011, the secondary industry proportion in most western resource-based cities exceeded the national average level. In addition, the proportion of the secondary industry in Karamay, Panzhihua, and Yan’an is as high as 70%, and they are all relatively mature cities for developing the secondary industry. Highly dependent on the development of the secondary industry is the main feature of the economy of western resource-based cities. However, after 2011, the proportion of the secondary industry in the 37 resource-based cities in the western region has been decreasing, showing a state of linear decline.

It can be analyzed that the proportion of the tertiary industry in most resource-based cities in the western region is still low, and generally lower than the national average level, indicating that the role of the tertiary industry in promoting the economy is not as good as that of the secondary industry in the economic growth of these resource-based cities. However, during the two decades from 2006 to 2020, the proportion of the tertiary industry in most resource-based cities has gradually increased, indicating that in the western resource-based regions, the secondary industry has no longer been the core driving force for economic growth, and the growing tertiary industry has promoted the improvement of the traditional industrial structure.

### 4.2 Level of industrial structure optimization

Considering the optimization of the industrial structure, in the whole industrial structure, the proportion of dominant industries has gradually evolved. The optimization of the industrial structure can show the resilience of the regional industrial structure. This paper uses the optimization index of the industrial structure to represent the optimization degree of the industrial structure level in the western resource-based regions to analyze the evolution of the industrial structure level in the western resource-based regions. Three time points of 2008, 2013, and 2019 are selected, and the Z-score method is used to preprocess the optimization index of the industrial structure. As the geometric interval classification method helps to ensure the same number of samples of all types and integrates the advantages of the natural break point method and quantile method, the optimization level of industrial structure in resource-based areas is divided into five gradients [48]: lower, low, medium, high, and higher, as shown in the Table 5.

**Table 5:** Upgrading level of industrial structure in western resource-based regions.

City	2008	2013	2019
Baotou	medium	high	high
Wuhai	low	low	lower
Chifeng	low	medium	higher
Erdos	medium	medium	lower
Hulunbeier	high	high	high
Baise	low	low	low
Hechi	medium	high	high
Hezhou	lower	higher	higher
Guangyuan	high	higher	low
Nanchong	medium	low	low
Guangan	higher	low	higher
Zigong	medium	low	medium
Luzhou	low	medium	higher
Panzhihua	lower	low	higher
Dazhou	medium	low	medium
Ya’an	low	low	higher
Liupanshui	lower	medium	higher
Longnan	high	high	high
Shizuishan	lower	lower	low
Anshun	high	high	high
Qujing	low	higher	medium
Baoshan	high	high	higher
Zhaotong	higher	low	medium
Lijiang	high	high	high
Lincang	higher	medium	high
Yan’an	lower	lower	lower
Tongchuan	medium	medium	higher
Weinan	higher	higher	medium
Xianyang	higher	medium	low
Baoji	low	lower	lower
Yulin	lower	lower	lower
Baiyin	low	medium	medium
Wuwei	high	higher	high
Zhangye	higher	high	high
Qingyang	lower	lower	lower
Pinliang	higher	high	high
Kelamayi	lower	lower	lower

The following observations are made from the table. Firstly, the evolution of the industrial structure in the western resource-based regions accelerated from 2008 to 2013, and the optimization level of the industrial structure in most regions improved. Among them, Baotou and Hechi moved from a middle grade to a high grade in 2008, and Zhangye and Pingliang moved from a high grade to a higher grade in 2008. Thus, the industrial structure is optimized. Chifeng, Luzhou, Liupanshui, Hezhou, Qujing, and Baiyin have all dropped out of the low-level sequence. The distribution of industries has gradually improved, and the resilience of the industrial structure has strengthened.

Second, the optimization of the industrial structure showed a steady improvement from 2013 to 2019. The number of resource-based regions with high-level industrial structures has increased to 10, and the number of regions with relatively high-level industrial structures has increased to 9. Among them, the industrial structures of Guang’an, Panzhihua, and Ya’an evolved rapidly, from a low level in 2013 to a high level in 2019, with strong upgrading ability of the industrial structure and further improvement of industrial structure resilience. The industrial structure has shown an upward evolution. In 2019, the number of regions with low or lower levels of industrial structure optimization was 12, with limited change. This is because the western resource-based regions have large individual differences and significant spatial imbalance characteristics. Some regions have successfully transformed and improved their industrial optimization level, while some regions have been at a low level for a long time. The level of industrial structure in a small number of areas has regressed.

Thirdly, in 2008, the optimization of the industrial structure in the western resource-based regions was different but did not show a significant convergence trend, indicating a large gap in the degree of the optimization of the industrial structure among regions. Among the 37 regions, 7 have a high level of industrial structure optimization, which shows that the industrial structure of these regions is better than that of other regions. There are 16 resource-based regions at lower and low levels, which means that the optimization and upgrading ability of the industrial structure in these regions is significantly behind that of other regions.

To summarise, although there are significant regional differences in the evolution of the optimization of the industrial structure from 2008 to 2019, most of the resource-based regions show a trend of improvement, and the evolution and adjustment of the industrial structure are “321,” which is crucial for the improvement of the resilience of the industrial structure. Among them, Hulunbuir, Anshun, Lijiang, Wuwei, Zhangye, Pingliang, and Longnan have always been at a relatively high level of industrial structure optimization. Wuhai, Baise, Baoji, Yilin, Qingyang, Shizuishan, and Karamay have been at a low level of industrial structure optimization for a long time, which indicates that the evolution speed of the industrial structure in these regions is relatively slow. The industrial transformation and upgrading need to be accelerated to improve the resilience of the industrial structures in these regions. From the perspective of the whole period, the number of low-grade and lower-grade regions has decreased, while the number of medium-grade, high-grade, and higher-grade regions has steadily increased.

### 4.3 Comprehensive evaluation of the resilience of the industrial structure

**Fig 2:**
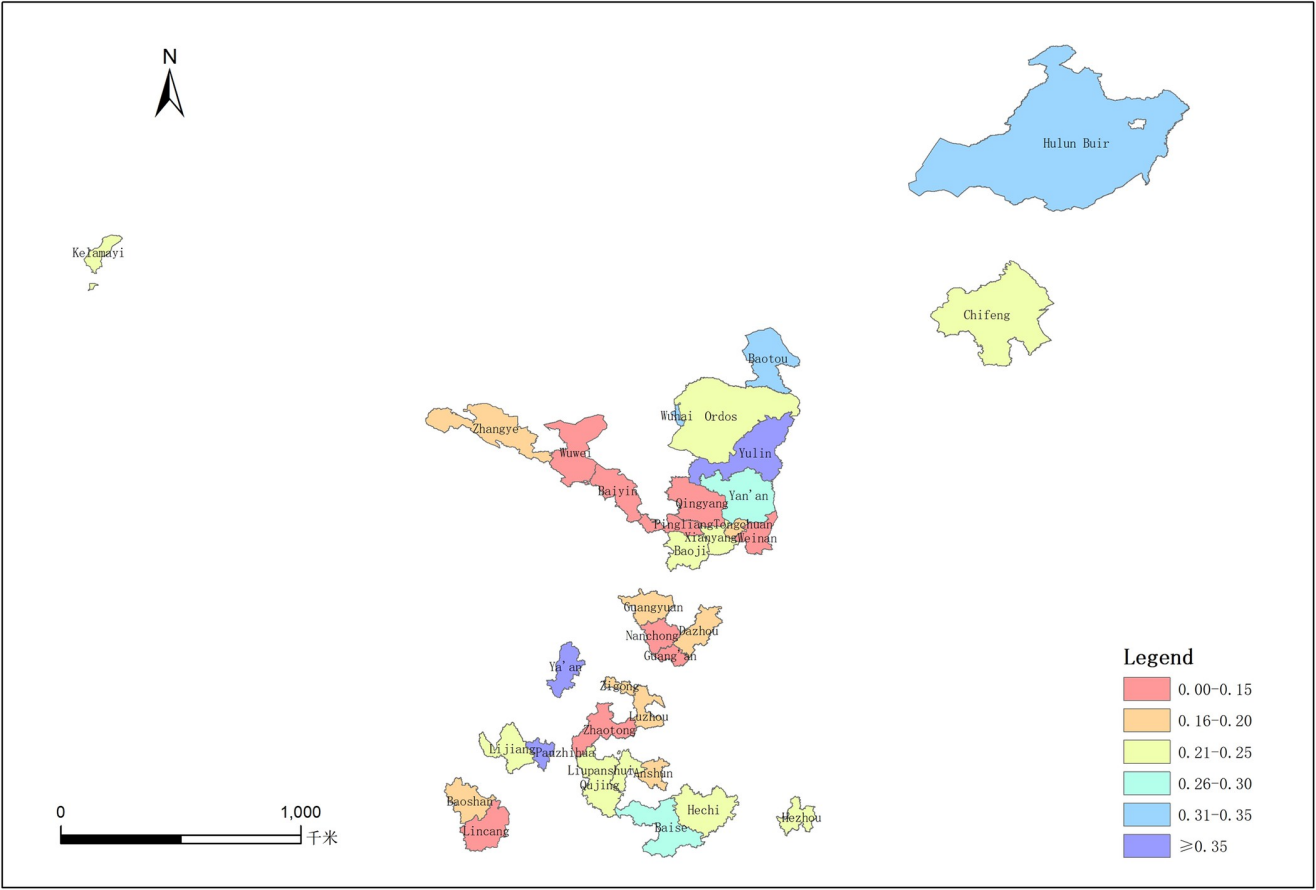
Comprehensive score of industrial structure resilience in western resource-based regions.

This paper selects the data from 2008, 2012, 2015, 2017, 2019, and 2021 in the western resource-based region for analysis, and calculates the score of the current situation of the industrial structure development in the western resource-based region. The indicators involved in the evaluation include the ability to resist risks, the ability to adapt to development, and the ability to innovate and transform; the calculated weights are 0.5588, 0.0733, and 0.3679, respectively. After calculation, the comprehensive score of the development status of the industrial structure in the western resource-based regions is shown in the Table 6 and Fig 2.

**Table 6:** Comprehensive score of industrial structure resilience in western resource-based regions.

City	2008	2012	2015	2017	2019	2021	AVERAGE
Tongchuan	0.10	0.17	0.19	0.22	0.24	0.21	0.18
Baoji	0.11	0.19	0.25	0.37	0.30	0.28	0.23
Xianyang	0.09	0.16	0.22	0.57	0.24	0.22	0.23
Weinan	0.07	0.13	0.15	0.22	0.18	0.16	0.14
Yan’an	0.17	0.28	0.27	0.24	0.36	0.32	0.25
Yulin	0.17	0.39	0.37	0.50	0.57	0.48	0.40
Baiyin	0.08	0.12	0.12	0.26	0.14	0.09	0.14
Zigong	0.09	0.16	0.17	0.20	0.24	0.21	0.16
Panzhihua	0.29	0.35	0.36	0.41	0.44	0.39	0.36
Luzhou	0.09	0.14	0.18	0.22	0.27	0.2	0.17
Guangyuan	0.10	0.15	0.18	0.20	0.26	0.19	0.17
Nanchong	0.07	0.11	0.13	0.16	0.19	0.15	0.13
Guangan	0.07	0.12	0.15	0.17	0.20	0.16	0.14
Dazhou	0.09	0.13	0.16	0.19	0.21	0.18	0.15
Ya’an	0.34	0.65	0.72	0.44	0.50	0.43	0.51
Liupanshui	0.13	0.15	0.27	0.39	0.27	0.15	0.23
Anshun	0.11	0.15	0.14	0.26	0.25	0.2	0.17
Qujing	0.09	0.12	0.15	0.39	0.28	0.17	0.19
Baoshan	0.04	0.07	0.22	0.27	0.30	0.26	0.23
Zhaotong	0.03	0.06	0.14	0.21	0.17	0.13	0.11
Lijiang	0.21	0.08	0.10	0.44	0.30	0.24	0.21
Lincang	0.03	0.07	0.09	0.31	0.25	0.19	0.14
Baotou	0.30	0.32	0.34	0.38	0.38	0.35	0.34
Wuhai	0.30	0.33	0.33	0.32	0.38	0.34	0.33
Chifeng	0.18	0.26	0.27	0.21	0.23	0.2	0.23
Erdos	0.27	0.22	0.12	0.26	0.27	0.22	0.23
Hulunbeier	0.31	0.30	0.32	0.32	0.34	0.31	0.32
Baise	0.24	0.31	0.32	0.37	0.18	0.11	0.26
Hezhou	0.22	0.25	0.14	0.28	0.33	0.23	0.24
Hechi	0.23	0.25	0.28	0.29	0.13	0.1	0.21
Wuwei	0.07	0.11	0.13	0.13	0.15	0.11	0.12
Zhangye	0.12	0.17	0.20	0.20	0.22	0.19	0.18
Pinliang	0.06	0.10	0.12	0.11	0.13	0.11	0.11
Qingyang	0.06	0.12	0.14	0.12	0.16	0.13	0.12
Longnan	0.12	0.14	0.16	0.16	0.18	0.15	0.15
Shizuishan	0.17	0.28	0.21	0.23	0.22	0.2	0.22
Kelamayi	0.27	0.22	0.09	0.28	0.26	0.21	0.22

In the Table 6, the mean value of the comprehensive score of the western resource-based regions from 2008 to 2021 is 0.21, the maximum value is 0.53, and the minimum value is 0.12, indicating that the overall industrial development level of the western resource-based regions is low, the resilience of the industrial structure needs to be strengthened urgently, and there are significant differences among regions. On the whole, in the stage of high-quality development, the western resource-based regions are generally faced with the problems of resource exhaustion, environmental degradation, and industrial contraction. Therefore, the resilience of the industrial structure is very weak, which is a hidden danger to China’s economic development. Driven by the strategy of western development and environmental protection, although the overall development level of western resource-based cities has gradually improved, compared with the cities in eastern and central China, the western resource-based cities are still at a lower level. They will remain at a relatively low level for a long time.

## 5. Conclusions and suggestions

This paper takes 37 resource-based cities in western China from 2008 to 2021 as the research object, comprehensively uses the entropy weight method, global spatial autocorrelation analysis, and industrial structure optimization index to analyze the resilience of industrial structure in western resource-based cities from two dimensions of time and space, and draws the following conclusions.

1. The heterogeneity of resource endowments and industrial structures in different regions determines the differences in the distribution of enterprises at each node of the industrial chain among regions. The industry resilience of western resource-based regions is weak, which reflects that the overall level of industrial development in the region during the study period is slowly improving. After the COVID-19 epidemic in 2020, the industrial chain has broken, and the transformation of the industrial structure is crucial to promote the improvement of industrial structure resilience.
2. In the face of the same impact, the resilience of the industrial structure of different regions is different. The improvement of industry-related diversification can help cities better withstand the impact of the COVID-19 pneumonia epidemic in 2020. The regions with a high proportion of secondary and tertiary industries show stronger resilience of the industrial structure in the face of crisis than other regions.
3. The improvement of the resilience of the industrial structure of western resource-based cities depends on the improvement of the supply quantity of resources and the supply rate of the market and the strengthening of the echelon allocation of the industrial structure. The strengthening of the resilience of the industrial structure is mainly controlled by the growth rate of regional GDP, per capita GDP, investment in science and technology, local financial supply level, and other factors.
4. The general trend of the development of today’s era is informatization, people’s yearning for a better life, and economic globalization, which respectively provide the feasibility in terms of technology, demand, and environment for building the resilience of China’s industrial chain, and provide a good policy environment for the resilience of forging and casting industrial chain. It is necessary to strengthen the resilience of the current industry from an all-round and multi-angle. To achieve three-dimensional monitoring and early warning of the risk of industrial chain breakage, coordination between industrial policies and industrial chain policies, improving the ability of chain breakage repair and regeneration, and finally achieving the goal of promoting the safety and modernization of industrial chain in western resource-based regions by improving the resilience of industrial chain under great changes is essential.
5. Through in-depth discussion on the resilience of the industrial structure in western resource-based regions, this study expands the research field and category of industrial resilience. It puts forward corresponding countermeasures for the high-quality development of resource-based cities. ① A scientific evaluation method is adopted to carry out cognitive evaluation on the positioning and life stage of resource-based cities to clarify the industrial development path within the specific stage; ② To reduce the degree of resource dependence as the primary goal, policy regulation and technology investment as the fundamental means, focusing on the improvement of residents, employment level and the derivation of emerging industries is essential; ③ Improving the allocation rate of resources and the efficiency of resource allocation in western resource-based areas are the inevitable requirements for accelerating the construction of a new economic development pattern, and also an important power source to achieve high-quality development; ④ Enhance industrial resilience and competitiveness from five aspects: strengthening industrial chain coordination, industrial agglomeration and integrated development, promoting industrial structural adjustment, enhancing scientific and technological innovation capacity, and embracing new technologies and new forms of business; ⑤ Promote the extension and diffusion of the industrial chain from the perspective of economic and technological links, give full play to the radiation effect of innovation ability and scale efficiency of manufacturing enterprises, explore a new mode of “resource +” on the industrial background to maintain competitiveness and ensure employment, and build a whole industrial chain system guided by institutional orientation based on characteristics and advantages, and linked by technological links.
